# Bibliographical Department

**Published:** 1880-05

**Authors:** 


### BIBLIOGRAPHICAL DEPARTMENT.

"Nothing extenuate, nor set down aught in malice."

"A Practical Treatise on Nervous Exhaustion, (Neurasthenia.) Its Symptoms, Nature, Sequences and Treatment." By George M. Beard, A. M., M. D., Fellow of the New York Academy of Medicine, of the New York Academy of Sciences, Vice-President of the American Academy of Medicine, Member of the American Neurological Association, of the American Medical Association, the Neurological Society, etc. William Wood & Company, publishers, 27 Great Jones Street, New York, 1880. We are indebted to the author for a copy of the foregoing work. We noticed the prospectus of the book in a previous issue of our journal, and expressed a decided opinion on what we expected the work would be, from our knowledge of Dr. Beard. The favorable opinion then given as to the probable character of the work, has been fully sustained by its perusal. To say that the book is absolutely faultless would be to state what could not be truthfully asserted of any production; but we feel warranted in expressing the opinion that no one will rise from its perusal without feeling under strong and lasting obligation to its intelligent author for the valuable information he has received. Indeed, it supplies practical knowledge that can be readily utilized in certain conditions of the nervous system that is not to be obtained in so small a compass elsewhere; and some facts, ideas and items to be found nowhere else in our vernacular literature. We warmly commend the book to our readers.

We have received from the Bureau of Medicine and Surgery, Navy Department, a copy of Hygienic and Medical Reports by medical officers U.S. Navy, 1879. Prepared for publication under the direction of the Surgeon-General of the Navy. By Joseph B. Parker, A. M., M. D., surgeon U. S. Navy, assistant to the Bureau of Medicine and Surgery. The volume contains 1079 pages, and embraces a large number of valuable articles written by various medical officers of the navy, on the topography and hygiene as well as the diseases usually prevalent at particular seasons, or developed under particular conditions and circumstances in various parts of the world. Drawings are introduced to illustrate the text wherever it is necessary they should be used for that purpose, *e. g.*, sections and parts of a ship are represented to show where certain occurrences related in the text took place, and they are thus rendered easy of comprehension to those unfamiliar with nautical verbiage. In several respects it is even a more valuable production than the "Medical History of the Late Civil War," published under the auspices of the head of the United States Army.

We are pleased to add the "North Carolina Medical Journal" to our list of exchanges. It is published at Wilmington, N. C., and edited by Drs. M. J. De Rosset and Thomas F. Wood. Annual subscription $3.00. The numbers before us bear evidence of ability and energy in getting them up, and the enterprise deserves the support of the profession.

We are glad to renew our acquaintance with the "Nashville Journal of Medicine and Surgery." The March issue, the only one received, is fully up to its predecessors in ante-bellum times. It is published in Nashville, Tennessee, and edited by Dr. C. S. Briggs, at $3.00, in advance.

Another member of the "Practitioner" family has greeted our sanctum, and comes with a bright and pleasant face, indicating clearly its willingness to receive us into the family circle, of which it is a junior member, though still a year older than ourself. We extend to our brothers Blackie, Roberts and Eve our best wishes for their editorial success, and a continuously increasing subscription list to the "Southern Practitioner." Published also at Nashville, Tennessee.

"Aspiration of the Knee Joint." By Henry O. Marcy, A. M., M. D., Cambridge, Mass., Vice-President of the American Medical Association; Member of the Massachusetts Medical Society; Fellow of the American Academy of Medicine; Boston Gynecological Society, etc. (From the author). We are glad Dr. Marcy has published the result of his aspirations of the knee-joint, as it will show the timid and inexperienced surgeon that there is really little, if any, danger from a procedure that is capable of affording so much relief in certain cases. We became fully convinced, as a surgeon during our late civil war, that the former teaching on the subject of opening the knee joint was entirely erroneous, and that little if any more danger was likely to result from exposure of the joint to the atmospheric air, *cæteris paribus*, than would be reasonably apprehended in any other similarly exposed part of the body. We were, in one instance, compelled to amputate at and through the knee-joint, and finding recovery took place as speedily and with as little untowardness as could have been expected in any other situation, did not hesitate to act in a similar manner, from choice, when the mutilation of the limb seemed to justify the procedure; and our recollection is the patients did well in every instance, and came out of the hospital with a better and more useful stump than could have resulted from any other operation.

We have received from Dr. Charles Kelsey a reprint of a paper from the "Hospital Gazette," read before the New York Clinical Society, on "Functional Heart Troubles," by himself, which we have examined with interest.

Our friends Dr. J. C. Le Hardy, of Savannah, Ga., and Robert B. S. Hargis, of Pensacola, Florida, have favored us with their respective views on yellow fever and the action of the National Board of Health in relation thereto, in sending their printed circulars on the subject. We would be glad to have more elaborate papers from these gentlemen on this most important matter, and, therefore, respectfully request them to prepare articles for publication in the pages of the INDEPENDENT PRACTITIONER.

We add the "New Orleans Medical and Surgical Journal" to our list of exchanges with much pleasure. It is edited by Drs. S. M. Bemiss, W. H. Watkins and S. S. Herrick, and is published in New Orleans, monthly, at $5.00 per annum, in advance. It is the best journal in the southwest.

The first number of the "Arkansas Medical Journal" for April has reached us. It is edited by Jonathan J. Jones, M. D., Little Rock, Arkansas. Subscription $3.00 a year, in advance. Single copies 30 cents. It is printed in clear type, and presents a very clean and neat appearance. We wish it success.

We are indebted to Dr. Edward C. Mann, Superintendent of Sunnyside, a private medical home for nervous invalids, Fort Washington, New York, for two well-written and unusually interesting little monographs. First, The Nature and Treatment of Inebriety; also the Opium Habit and its Treatment; second, Insanity: Its Etiology, Diagnosis, Pathology and Treatment, with Cases Illustrating Pathology, Morbid Histology and Treatment. The first consists of five papers, read before as many different bodies or societies interested in the subjects named in the contents above, and are worthy of perusal by any one. The second compresses, in terse and graceful diction, as much valuable practical information on insanity as one could expect to find in so small a space, and shows conclusively the author's familiarity with the subject on which he writes.

Dr. B. B. Browne, of this city, has sent us a reprint of a paper read by him before the Baltimore Clinical Society, February 6th, 1880, entitled, "Retention of the Placenta after Abortion," which, though brief, contains valuable hints and suggestions on the subject to which it relates.

The Anaesthetic Use of the Bromide of Ethyl. Dr. Levis has had extensive experience with the new anaesthetic, and from his high standing as a surgeon we regard his utterances on the subject entitled to the fullest confidence and respect of the profession.—Ed.

"The Importance of Preserving the Teeth," by Thos. L. Sydnor, D. D. S., Danville, Va. This little book is illustrated, and contains much which should be known by every one. It is intended for the laity and is highly commendable for its *multum in parvo*.

"Practical Information about the Teeth. A Book for the People," by Arthur Holbrook, D. D. S. Published by the Wisconsin State Dental Society. It is handsomely printed, illustrated, and worthy the careful attention of all. We are very glad to see this society make such an important move.

"The Dental News." Edited and published by T. P. Wagoner, Knightstown, Md. A monthly journal replete with well-selected matter. Per annum, $1.00.

Volume 1, No. 1, of "The Odontographic Journal," edited by J. E. Line, D. D. S., and published by Davis & Leyden, Rochester, is a neat and interesting quarterly journal. Annual subscription, $1.00.

We are indebted to our esteemed relative and friend, Dr. William A. Byrd, of Quincy, Ill., for a printed copy of the proceedings of the Adams County Medical Society, on "Laparotomy and Colotomy with Formation of Artificial Anus."

