# Erratum

**Published:** 1880-06

**Authors:** 


ERRATTUM.—The Dental News is published at Knightstown, Indiana, not Md., as the devil made it.

